# Primary Healthcare Solo Practices: Homogeneous or Heterogeneous?

**DOI:** 10.1155/2014/373725

**Published:** 2014-01-12

**Authors:** Raynald Pineault, Roxane Borgès Da Silva, Sylvie Provost, Marie-Dominique Beaulieu, Antoine Boivin, Audrey Couture, Alexandre Prud'homme

**Affiliations:** ^1^Direction de Santé Publique de l'Agence de la Santé et des Services Sociaux de Montréal, 1301 rue Sherbrooke Est, Montréal, QC, Canada H2L 1M3; ^2^Institut National de Santé Publique du Québec, 945 Avenue Wolfe, Québec, QC, Canada G1V 5B3; ^3^Centre de Recherche du Centre Hospitalier de l'Université de Montréal, 3480 rue Saint-Urbain, Hôtel-Dieu (Pavillon Masson), Montréal, QC, Canada H2W 1T8; ^4^Faculté des Sciences Infirmières, Université de Montréal (Pavillon Marguerite d'Youville), CP 6128, Succursale Centre-ville, Montréal, QC, Canada H3C 3J7; ^5^Département de Médecine Familiale, Université de Montréal (Pavillon Roger-Gaudry), 290 Boulevard Édouard-Montpetit, Bureau S-711, Montréal, QC, Canada H3T 1J4; ^6^Centre de Recherche du CSSS Champlain-Charles-Le Moyne, 150 Place Charles-Lemoyne, Bureau 200, Longueuil, Montréal, QC, Canada K4K 0A8

## Abstract

*Introduction*. Solo practices have generally been viewed as forming a homogeneous group. However, they may differ on many characteristics. The objective of this paper is to identify different forms of solo practice and to determine the extent to which they are associated with patient experience of care. *Methods*. Two surveys were carried out in two regions of Quebec in 2010: a telephone survey of 9180 respondents from the general population and a postal survey of 606 primary healthcare (PHC) practices. Data from the two surveys were linked through the respondent's usual source of care. A taxonomy of solo practices was constructed (*n* = 213), using cluster analysis techniques. Bivariate and multilevel analyses were used to determine the relationship of the taxonomy with patient experience of care. *Results*. Four models were derived from the taxonomy. Practices in the “resourceful networked” model contrast with those of the “resourceless isolated” model to the extent that the experience of care reported by their patients is more favorable. *Conclusion*. Solo practice is not a homogeneous group. The four models identified have different organizational features and their patients' experience of care also differs. Some models seem to offer a better organizational potential in the context of current reforms.

## 1. Introduction

Recent reforms in healthcare delivery have greatly modified primary care medical practice, by fostering the grouping of physicians into more complex and large organizations [1]. Consequently, the number of solo practices has decreased considerably. In Canada, the percentage of physicians in solo practice was estimated at 51.8% in 1986-1987 [2]. In 1997, this figure had decreased to 31.3% and in 2010 to only 22.3%. Male and older physicians were proportionally overrepresented in this mode of practice [2–5]. This trend has also been observed in other countries, namely, in The Netherlands where the percentage of solo practitioners decreased from 67.4% in 1990 to 39.1% in 2010, while group practice increased proportionally [6, 7]. A decrease in small practices has also been reported in the UK [8].

Echoing this trend, solo medical practice has been considered obsolete [9]. However, analysts are not unanimous, as many contend that small practices, including solo practices, must be maintained [10]. Solo practice is cherished by many doctors, because it fosters greater professional autonomy, a core value of the medical profession [1, 11]. Networking, either through formal collaborative agreement or patients' referral and affiliation with other practice settings becomes an essential ingredient for smaller size and particularly solo practices to hinder professional isolation [12, 13].

Solo practice has generally been studied from the angle of its size, as measured by the number of physicians it comprises. Studies have examined the relationship between size of practice and various measures of utilization, experience or quality of care. Overall, these studies report a favorable experience of care, high degree of satisfaction, and appropriate use of services among patients treated by solo physicians [6, 14–16]. However, some agencies responsible for professional development among their members have expressed concerns about isolated solo physicians having limited interactions with their colleagues [12, 17].

Such isolation is not generalized. Solo physicians do not necessarily constitute a homogeneous group, precisely because of the different networking strategies they adopt [13, 18]. For instance, physicians in rural areas are often constrained to solo practice, but they make up for this isolation by exchanging and collaborating with other primary healthcare and specialists' practices [19, 20]. In sum, different forms of solo practice can result from linkage with exchange and collaboration networks, resources on-hand, and affiliation of physicians with other organizations. In turn, these different forms of practice may influence patient experience of care [2].

This paper aims to identify the different forms of solo practice in two regions of Quebec, Montréal and Montérégie, in 2010 and to determine the extent to which they are associated with variations in patient experience of care.

## 2. The Current Reform of PHC Organization in Quebec

In the early 2000s, Québec initiated important reforms of its healthcare system. Family Medicine Groups (FMGs) and Network Clinics (NCs) were created with the aim of improving continuity, integration of care, and accessibility of services. As of October 2013, there were 254 FMGs out of the 300 targeted. A complementary PHC organizational model, the Network Clinic, is being currently implemented. This PHC model aims at fostering accessibility through walk-in visits and providing access to specialists and to technical support services, such as X-rays and lab tests. Their creation was initiated by the Montréal Regional Health Agency as a complement to FMGs, in response to requests by the regional medical association. The healthcare reform also included the creation of health and social services centers (HSSCs), merging acute care hospitals, long-term care hospitals, and local community health centers (CLSC) on a geographical basis. In addition to their responsibility for providing health services to the population of their territory, HSSCs were mandated to lead the implementation of local services networks, notably by fostering collaboration involving PHC organizations, and to support the implementation of FMGs.

## 3. Methods

### 3.1. Research Design

Our study consisted of two surveys conducted in 2010 in the two most populous regions of Quebec, Montréal and Montérégie which represent more than 40% of the province's total population [21]. The first was a population-based telephone survey involving 9180 randomly selected adults (aged 18 years or older). The sample was nonproportionally stratified (about 400 respondents in each of the 23 health and social services centre territories). Response rate was 56% [22]. Data were weighted by attributing the inverse probability of selection of participants in order to account for unequal sampling probabilities resulting from the stratified two-stage sampling (local area sampling and intrahousehold selection). In addition, a poststratification weighting was applied for age and sex distribution compared to census data. The second, a mail survey, involved all PHC organizations in the two regions (*n* = 606). Response rate was 62% [23]. In each organization, a key informant, usually the doctor responsible for the practice's professional and administrative matters, completed the questionnaire. Since we had basic information on all organizations, including the nonresponding ones, we applied an imputation technique to nonresponding organizations, based on the probability of responses, given the region, type, and size of the practice group to which they belonged [24]. The two surveys were linked through identification by respondents to the population questionnaire of their regular source of PHC services. Among the 606 organizations, we identified 213 solo practices from which 187 were named at least once as usual source of care by 743 patients from the population survey. Categorization as solo practice was based on the following criteria:

- only one doctor on site;
- unique civic address or, if in a building with other doctors, a unique suite or office number;
- unique and distinct telephone number;
- no sharing of resources with other doctors on the premises (office space, personnel, medical charts, etc.).

The study was carried out according to the principles of the Helsinki Declaration. The research Ethics Committee of the Agence de la Santé et des Services Sociaux de Montréal approved the study. Participants had to sign informed consent forms and were told that they could withdraw any time during the study.

### 3.2. The Population Questionnaire

The population questionnaire assessed respondents' current affiliation with PHC organizations, their health services utilization profiles, attributes of their care experience, and their reported unmet needs [25]. Construction of the questionnaire drew upon various validated instruments for assessing experience of care, especially the one used in the primary care assessment survey (PCAS), and the primary care assessment tool (PCAT) [26–28]. We adapted questions extracted from these questionnaires to the context of our study and added a few others when a topic was not addressed. For example, none of these tools had addressed geographic or temporal accessibility to usual source of care [29]. Therefore, we developed questions pertaining to this important aspect of our study. The Institut de la statistique du Québec eventually included many of our questions on experience of care in a large-scale population survey of Quebec's population conducted in 2010-2011, and which extended the validation process [30].

We selected 21 indicators of experience of care and grouped them under the four following dimensions: accessibility (6), continuity (5), comprehensiveness (5), and care outcomes (5) [21]. A factor analysis was then carried out with the Varimax Orthogonal option and internal consistency of the scales tested. Cronbach alpha was 0.63 for continuity, 0.79 for comprehensiveness, and 0.82 for care outcomes. For accessibility, we included items related to various aspects of this concept (accommodation, travel time, etc.). Because of their heterogeneity and the fact that those items were more causal than effect indicators, we considered that these grouped indicators constituted a composite formative index rather than a reflective scale [31]. Hence, we did not submit them to factor analysis which is an inappropriate method of analysis in the formative approach since items are not necessarily correlated [32].

Aside from information on experience of care, the population questionnaire contained information on use of services, reporting of unmet needs, presence of morbidities, and preventive care received, as well as sociodemographic characteristics of respondents [21, 25].

### 3.3. The Organization Questionnaire

The structure of the organization questionnaire was based on four core elements of organizations. Vision refers to goals, values, and orientations shared by members of an organization; resources concern availability, quantity, and types of resources that can be mobilized by the organization's members; structure formalizes rules of governance, conventions, and procedures that regulate the behavior of organizational actors, and practices relate to coordination, administrative, and professional mechanisms that underpin service delivery [21, 23, 33].

The questions stem from various sources [34–36]. However, most of the questions were formulated by the study authors, in accordance with the framework presented above. A first validation of the questionnaire was performed during the first 2005 study [16]. An expert group was consulted to assess exhaustivity and relevance of the questions. A pretest was then carried out among PHC organizations located in geographical areas other than the two regions under study. It is interesting to note that this questionnaire was the principal source for a Canadian Institute of Health Information questionnaire aimed at documenting the composition of Canadian PHC organizations [37].

Because of the type of indicators generated from the questions, notably their objective and descriptive (reporting) rather than evaluative (rating) character, they were considered to form composite formative indices rather than metric reflective scales [31, 32]. Factor analysis is not appropriate in this case since indicators of a same index are not necessarily correlated.

### 3.4. Active Independent Variables

The following indicators characterizing solo practices were used as active variables for constructing a taxonomy: presence of a nurse; presence of specialist doctors and other professionals in the building; availability of information technology; access to X-ray services or blood sample collection in the building; collaboration agreement with other PHC clinics; collaboration agreement with a hospital; and doctor's affiliation with other PHC clinics, emergency rooms, or hospitals. These active variables served to construct a taxonomy of solo practices, using a multiple correspondence analysis associated with a hierarchical ascending classification [18, 38–40].

### 3.5. Illustrative Variables

In addition to the active independent variables, the following illustrative variables were used to further characterize the classes of the taxonomy: age and sex of physicians, presence of other GPs in the building, time spent in the clinic (26 hours/week or more), scope of services provided, and acceptance of new patients.

### 3.6. Dependent Variables

Dependent variables are related to experience of care and utilization of services reported by population survey respondents. Experience of care variables are accessibility, continuity, comprehensiveness, care outcomes, reporting of unmet needs, and having a family doctor. Details concerning operationalization of these variables are presented in Table 1.

### 3.7. Control Variables

In analyzing the relationships between taxonomy and experience/utilization of care, we controlled for the following variables, all derived from the population survey: age and sex of respondents, economic status, perceived health status, and presence of morbidities. Operationalization of these variables is itemized in Table 2.

### 3.8. Data Analysis

We used two statistics to describe the taxonomy: value test and Cramer V coefficient. The value-test indicates the importance of a variable in constructing that taxonomy. It measures the distance between the mean of all observations and the mean of the class, expressed by the number of standard deviations of a normal distribution [41]. Positive-value tests correspond to positive associations and negative-value tests correspond to negative associations. Cramer V coefficients are used to show the strength of associations between each variable employed in constructing the taxonomy and classes of taxonomy. Cramer V is considered weak when smaller than 0.30, medium between 0.30 and 0.49, and strong when at 0.50 or more [42]. The relationship between the taxonomy models and patient experience of care is shown first in bivariate analyses. Test of difference of proportions and Fisher's least significant difference test (LSD) for comparing means were performed. Results are then presented for multilevel, linear, or logistic regression analyses, depending on the form of the dependent variables, controlling for patients characteristics.

## 4. Results

### 4.1. Taxonomy of Solo Practices

Four models emerged from the taxonomy of solo practices (Table 3).

The model called “resourceless isolated” includes 79 practices (37.1%). It is characterized as having few resources in terms of availability of a nurse, other health professionals, information technologies, and X-ray and blood sample collection available on the premises. Moreover, level of collaboration of these practices is weak, both with other PHC practices and particularly with other healthcare organizations.

The 53 (24.9%) practices in the “resourceless collaborative” model also have few resources, but they have established collaborative relationships with other PHC practices and with hospitals. Doctors concentrate their activities in their main solo practice setting. In sum, these practices have probably compensated for their limited resources by establishing outside collaborations, to widen the range of services offered to their patients.

The 57 (26.8%) practices in the “resourceful not exclusive” model are characterized by their many resources, with their doctors likely to be affiliated with other PHC practices. These practices have not established collaborative relationships with other PHC practices and hospitals.

The fourth model is called “resourceful networked” and includes only 24 practices (11.3%). They show a high level of collaboration with other PHC practices and hospitals and have more resources. In addition, doctors tend to be affiliated with hospitals and emergency departments. These multisite activities, coupled with collaborative relationships with other practice settings, contribute to their high degree of networking.

Illustrative variables further characterize these four models. A high percentage of doctors in all four models spend 26 hours or more a week in their main practice, with the highest being in the “resourceful networked” (91.7%) and the lowest in the “resourceful not exclusive” model (77.5%). Practices in these two models are more likely to be located in buildings where there are other GPs and their doctors tend to be younger. Finally, these practices tend to offer a wide range of services (85%).

The degree of association between the four models and individual variables that served to construct the taxonomy is measured using Cramer's V coefficient (Table 3). Five variables have strong relationships with the models: collaboration agreement with another medical practice and with a hospital, availability of X-ray services and blood sample collection in the same building, presence of a nurse in the practice, and presence of a specialist or other health professionals in the same building. Among the four models, differences are greater between practices of the resourceless models (isolated and collaborative) and those of the resourceful model (nonexclusive and networked).

### 4.2. Patient Characteristics

The four models of the taxonomy are not different regarding their patients' characteristics. Patients of the “resourceless isolated” model differ in two characteristics: percentage of women and economic situation (Table 4).

### 4.3. Relationships of Care Experience with Models of the Taxonomy

Patients whose usual source of care is a “resourceful networked” practice report a better experience of care than those of the “resourceless isolated” practices. In addition, patients in the “resourceless isolated” practices report less continuity than those in the three other models (Table 5). Results of the multiple regression analysis confirm the better performance of “resourceful networked” practices compared to “resourceless isolated” practices regarding accessibility and outcomes of care and, to a lesser degree, continuity (Table 6).

## 5. Discussion

The main result of this study is that solo practices do not form a homogeneous group. The four models presented differ on many characteristics, particularly the “resourceless isolated” and the “resourceful networked” models. To our knowledge, very few studies have addressed this question, solo practices being generally viewed as the lowest category on a scale of practice size. More specifically, three types of characteristics distinguish the models of the taxonomy: collaborative relationships of the practice with other PHC practices and hospitals, affiliation of doctors with other practice settings, and level of resources available. Two models are relatively resourceful and two are rather resourceless. Likewise, two models have established collaboration agreements with other PHC practices and hospitals, whereas the two other models have little or no collaboration. Physicians tend to have hospital affiliations in two models. Practices in the “resourceful networked” model have successfully established collaboration with other practices and doctor affiliation with hospitals; they also benefit from an appropriate level of resources. This model includes only 24 practices, whereas they are most prevalent (79) in the “resourceless isolated” model. This model representing 37.1% of the solo practices probably contributes to the poor reputation sometimes associated with solo practices. Since nearly a third of physicians in this group are aged 65 and over, these practices will likely incur severe losses in the near future. Practices in the “resourceless collaborative” model will be facing similar problems as 38% of their doctors are 65 and over (Table 3). In contrast, practices in the “resourceful not exclusive” and “resourceful networked” models have, respectively, 19.3% and 12.5% of physicians aged 65 and more. This demographic peculiarity seems to foresee a promising future for these practices.

The practices in the “resourceful networked” model yield better results regarding their patients' experience of care, compared with the practices in the “resourceless isolated” model.

The results concerning solo practices must be regarded in the light of current reforms that have taken place in PHC organizations in Canada. They raise the question of whether the new PHC models, based on group of increasing numbers of physicians on single sites within large multidisciplinary teams of health professionals, are the solution to problems faced in the delivery of PHC services [43]. The creation of new models of PHC service delivery, such as Family Medicine Groups (FMGs) and Network Clinics (NCs) in Quebec, adds resources and widens the range of services offered to a large population [44]. However, in the Montréal and Montérégie regions, almost 80% of PHC practices are not directly affected by the current reform, which is centered on creating new models [45, 46]. The stringent criterion of minimal number of physicians required to be eligible for FMG status is an obstacle that prevents many PHC practices from acquiring this status, regardless of their performance which, in certain cases, is higher than that of well-established FMGs [44].

To meet population needs, alternatives to the strategy of concentrating efforts and resources on a few “organizational champions” must be envisaged. This point was well expressed by Wensing et al. in conclusion to a study of eight European countries [6]: “Maybe GPs should organize themselves in networks of small practices rather than large clinics with many health care professionals. The organizational development of general practice should not only be determined by the professional perspective, but by patients' needs and preferences.”

A similar conclusion was reached by Smith et al. in a recent report of the King's Fund [8]: “…fundamental changes to the organizations and delivery of general practice and primary care become necessary. These include the linking together of practices in federation, networks or merged partnerships, in order to increase their scale, scope and organizational capacity. This will need to be done while preserving the local small scale points of access to care that are valued highly by patients.”

In light of the taxonomy just presented, solo practices in two models seem to be better prepared to face the challenges posed by such changes: these are practices in the “resourceful networked” and, to a lesser degree, of the “resourceful not exclusive” model. The former present conditions required for integration into larger networks and partnerships; the latter have established collaborative links with other PHC practices and hospitals through formal agreements and their doctors' affiliation.

## 6. Limitations and Strengths

The main limitation of the study is the relatively small number of PHC units (*n* = 213) and of patients attached to them (*n* = 743). It must be noted that in the case of PHC practices, the units' total population has been studied and not just a sample. Nevertheless, the small number of patients drawn from the population sample reduces the power of statistical analyses.

In spite of this limitation, interesting conclusions can be reached about the relationship of the models of the taxonomy with patient experience of care. These conclusions would need to be tested on larger populations. The nominal link between respondent in the population survey and usual of source of care in the organizational survey provides the opportunity to establish the relationship of patient experience of care with characteristics of their usual source of care. This is an original feature of the study.

## 7. Conclusion

Far from being homogeneous, solo practice takes on different heterogeneous forms and configurations. The taxonomy of solo practices has identified four models: “resourceless isolated,” “resourceless collaborative,” “resourceful not exclusive,” and “resourceful networked.” The practices in these four models present characteristics that distinguish them with regard to type and level of resources available and their relationships with other PHC practices and hospitals. Resourceful and networked practices through collaborative agreements and affiliation of their doctors with other practices seem to offer greater organizational potential to address challenges posed by currently ongoing PHC reforms.

## Figures and Tables

**Table 1:** Questions used for constructing experience of care indices.

Accessibility		Comprehensiveness	
At this place, if the doctor who is responsible for your care is not available, you can see another doctor	(0) Never	At this place, all your health problems are taken care of, whether they are physical or psychological	(0) Not at all agree
	(1) Sometimes		(1) A little
	(2) Often		(2) Somewhat
	(3) Always		(3) Strongly agree

At this place, how long does it take to see the doctor by appointment?	(0) 4 months or more	At this place, during your visits, the doctor takes the time to talk to you about prevention and asks you about your lifestyle habits	(0) Not at all agree
	(1) From 1 to 3 months		(1) A little
	(2) From 2 to 4 weeks		(2) Somewhat
	(3) Less than 2 weeks		(3) Strongly agree

How long does it usually take to get there?	(0) More than 30 minutes	At this place, they help you get all the health care services you need	(0) Not at all agree
	(1) From 15 to 30 minutes		(1) A little
	(2) Less than 15 minutes		(2) Somewhat
			(3) Strongly agree

At this place, the office hours are convenient	(0) Not at all agree	At this place, your opinion and what you want are taken into account in the care that you receive	(0) Not at all agree
	(1) A little		(1) A little
	(2) Somewhat		(2) Somewhat
	(3) Strongly agree		(3) Strongly agree

It is easy to reach someone at this place by telephone to make an appointment	(0) Not at all agree	At this place, you are given help to weigh the pros and cons when you have to make decisions about your health	(0) Not at all agree
	(1) A little		(1) A little
	(2) Somewhat		(2) Somewhat
	(3) Strongly agree		(3) Strongly agree

It is easy to talk to a doctor or nurse by telephone when this place is open	(0) Not at all agree
	(1) A little
	(2) Somewhat
	(3) Strongly agree

Continuity		Outcomes of care	

When you go to this place, you see the same doctor	(0) Never	The services you get there help you to better understand your health problems	(0) Not at all agree
	(1) Sometimes		(1) A little
	(2) Often		(2) Somewhat
	(3) Always		(3) Strongly agree

How long have you been going to this place?	(0) Less than 2 years	The services you get there help you to prevent certain health problems before they appear	(0) Not at all agree
	(1) From 2 to 5 years		(1) A little
	(2) More than 5 years		(2) Somewhat
			(3) Strongly agree

At this place, your medical history is known	(0) Not at all agree	The services you get there help you to control your health problems	(0) Not at all agree
	(1) A little		(1) A little
	(2) Somewhat		(2) Somewhat
	(3) Strongly agree		(3) Strongly agree

At this place, they are aware of all the prescribed medications you take	(0) Not at all agree	The professionals you see there encourage you to follow the treatments prescribed	(0) Not at all agree
	(1) A little		(1) A little
	(2) Somewhat		(2) Somewhat
	(3) Strongly agree		(3) Strongly agree

At this place, you can receive routine ongoing care for a chronic problem	(0) Not at all agree	The professionals you see there help motivate you to adopt good lifestyle habits	(0) Not at all agree
	(1) A little		(1) A little
	(2) Somewhat		(2) Somewhat
	(3) Strongly agree		(3) Strongly agree

**Table 2:** Patients' characteristics categories.

Variables	Categories
Age	18 to 29
	30 à 44
	45 à 64
	65 or more

Sex	Woman
	Man

Level of education	No diploma (elementary school)
	High school diploma
	College diploma (CEGEP)
	University degree

Economic situation	Very unfavorable
	Unfavorable
	Favorable
	Very favorable

Perceived health	Poor
	Average
	Good
	Very good
	Excellent

Morbidities	No risk factor* nor chronic disease**
	One risk factor or more but no chronic disease
	One chronic disease (with or without risk factor)
	Two chronic diseases or more (with or without risk factor)

*Risk factor includes high blood pressure, diabetes, and high level of cholesterol.

^∗∗^Chronic disease includes angina, infarct, arrhythmia, prior heart surgery, heart failure, pleural effusion or other heart problems, cerebrovascular disease, chronic bronchitis, emphysema or chronic obstructive lung disease, asthma, and rheumatism or arthritis.

**Table 3:** Organizational characteristics associated with the four solo practice models of the taxonomy.

Active independent variables	Resourceless isolated		Resourceless collaborative		Resourceful not exclusive		Resourceful networked		All models	Cramer V
	*n* = 79		*n* = 53		*n* = 57		*n* = 24		*n* = 213
	%	Value test	%	Value test	%	Value test	%	Value test	%
At least one nurse in the practice	2.5	−5.5	5.7	−3.2	43.9	4.5	62.5	4.5	21.1	0.558
Specialists or other health professionals in the building in which practice is located	26.6	−4.3	18.9	−4.6	82.5	6.4	83.3	3.8	46.0	0.580
At least one information technology available	34.2	−2.5	37.7	−1.2	63.2	2.9	62.5	1.5	46.0	0.268
Radiology or blood sample collection available in the building where practice is located	3.8	−6.7	5.7	−4.6	70.2	7.6	66.7	3.9	29.1	0.691
Collaboration with other PHC practices	1.3	−6.8	66.0	7.3	0.0	−5.9	75.0	5.3	25.4	0.754
Collaboration with hospitals	2.5	−7.0	64.2	6.2	7.0	−4.4	87.5	6.2	28.6	0.728
Doctor's affiliation with another PHC practice	19.0	−0.3	3.8	−3.7	36.8	3.6	25.0	0.2	20.7	0.297
Doctor's affiliation with another health facility	12.7	−2.0	20.8	−0.5	24.6	1.2	33.3	1.9	22.5	0.106

Illustrative variables	Resourceless isolated		Resourceless collaborative		Resourceful not exclusive		Resourceful networked		All models	Cramer V
	*n* = 79		*n* = 53		*n* = 57		*n* = 24		*n* = 213
	%		%		%		%		%

Age of doctor (≥65 years)	32.9		37.7		19.3		12.5		28.2	0.199
Sex of doctor (women)	34.2		26.4		24.6		29.2		29.1	0.094
Other general practitioners in the same building	15.0		16.7		45.0		33.3		27.6	0.306
Doctor works 26 hours/week or more in the clinic	82.5		83.3		77.5		91.7		81.9	0.107
Scope of services provided (broad or medium)	58.2		67.9		86.0		83.3		70.9	0.260
Doctor accepts new patients	62.5		58.3		70.0		83.3		66.4	0.128

**Table 4:** Characteristics of patients by solo practice model.

	Resourceless isolated	Resourceless collaborative		Resourceful not exclusive		Resourceful networked	
	(*n* = 296)	(*n* = 176)		(*n* = 173)		(*n* = 98)	
	%	%	*P*	%	*P*	%
Age (≥65 years)	24.2	19.3	0.197	24.3	0.991	28.9	0.414
Sex (women)	50.3	52.8	0.599	60.7^#^	0.028^#^	50.0	0.954
Level of education (high school degree or less)	43.6	41.5	0.677	45.9	0.592	51.0	0.192
Economic situation (unfavorable)	43.8	39.8	0.376	34.1^#^	0.033^#^	41.3	0.642
Perceived health (average or poor)	15.2	17.0	0.590	15.5	0.915	21.6	0.164
Morbidities (at least one chronic disease)	35.7	36.6	0.847	40.8	0.271	39.2	0.540

Reference: ^#^resourceless isolated (*P* ≤ 0.05).

**Table 5:** Mean scores (0–10 scale) of patients' experience of care by solo practice model.

	Resourceless isolated	Resourceless collaborative		Resourceful not exclusive		Resourceful networked	
	(*n* = 296)	(*n* = 176)		(*n* = 173)		(*n* = 98)	
	Mean	Mean	*P*	Mean	*P*	Mean
Accessibility	6.16	6.15	0.924	6.38	0.137	6.66^#^	0.006^#^
Continuity	8.74	9.12^#^	0.004^#^	9.23^#^	0.000^#^	9.29^#^	0.000^#^
Comprehensiveness	8.43	8.54	0.553	8.64	0.631	8.93^#^	0.029^#^
Outcomes of care	8.73	8.88	0.344	9.00	0.104	9.32^#^	0.003^#^

Reference: ^#^resourceless isolated (*P* ≤ 0.05).

**Table 6:** Relationships between patients' experience of care and solo practice models*.

(Score on 0–10 scale)	Resourceless isolated (*n* = 296)	Resourceless collaborative (*n* = 176)		Resourceful not exclusive (*n* = 173)		Resourceful networked (*n* = 98)	
		Coeff.	*P*	Coeff.	*P*	Coeff.	*P*
Accessibility	Reference	0.033	0.901	0.152	0.431	0.531^#^	0.039^#^
Continuity	Reference	0.116	0.560	0.244	0.236	0.320	0.101
Comprehensiveness	Reference	−0.038	0.879	0.038	0.893	0.359	0.189
Outcomes of care	Reference	0.028	0.899	0.084	0.734	0.580^#^	0.004^#^

Reference: ^#^resourceless isolated (*P* ≤ 0.05).

*Data adjusted for patients' age, sex, level of education, economic situation, perceived health, and morbidities.
